# The Turkish Pain Catastrophizing Scale-Child in Adolescents with Familial Mediterranean Fever: A Psychometric Analysis

**DOI:** 10.5152/ArchRheumatol.2025.11043

**Published:** 2025-05-29

**Authors:** Devrim Can Sarac, Deniz Bayraktar, Ozge Altug Gucenmez, Balahan Bora, Sema Savci, Sevket Erbil Unsal

**Affiliations:** 1 Department of Physiotherapy and Rehabilitation, İzmir Katip Çelebi University Faculty of Health Sciences, İzmir, Türkiye; 2 Clinic of Pediatric Rheumatology, Health Sciences University, İzmir Dr. Behçet Uz Pediatric Diseases and Surgery Training and Research Hospital, İzmir, Türkiye; 3 Division of Pediatric Rheumatology, Department of Pediatrics, Dokuz Eylül University Faculty of Medicine, İzmir, Türkiye; 4 Department of Physiotherapy and Rehabilitation, Acıbadem University Faculty of Health Sciences, İzmir, Türkiye

**Keywords:** Auto-inflammatory diseases, reliability, validity

## Abstract

**Background/Aims:**

Currently, no validated method exists to assess possible pain catastrophizing in adolescents with FMF. Thus, the objective of this study was to determine the validity and reliability of the Turkish Pain Catastrophizing Scale-Child (PCS-C) in adolescents with FMF by investigating internal consistency, test-retest reliability, and convergent validity.

**Materials and Methods:**

Turkish PCS-C, Visual Analog Scale during rest (VAS-rest) and activity (VAS-activity), and Pediatric Quality of Life Inventory (PedsQL) Arthritis Module 3.0 were administered to 66 adolescents with FMF (age: 13-18 years). Cronbach’s alpha value was calculated for internal consistency. Turkish PCS-C was re-administered 2 weeks later by phone to 24 participants to calculate intraclass correlation coefficients (ICCs) for determining test-retest reliability. Convergent validity was investigated by calculating Spearman’s Rank Correlation Coefficients (r_s_) between Turkish PCS-C and other evaluated parameters.

**Results:**

Item 8 was excluded from the Turkish PCS-C due to its low contribution to internal consistency. Following this, the Cronbach’s alpha was calculated as 0.934. The remaining items contributed to the total score (item-total correlations > 0.4), and Cronbach’s alpha did not differ significantly with the exclusion of any items (change < 1%). The Turkish PCS-C demonstrated excellent test-retest reliability (ICC = 0.925). Fair to moderate positive correlations were detected between the Turkish PCS-C total score and VAS-rest (r_s _= 0.373), VAS-activity (r_s _= 0.536), and PedsQL scores (r_s _= 0.551).

**Conclusion:**

Turkish PCS-C may be used as a valid and reliable tool to assess pain catastrophizing in adolescents with FMF.

· Unpredictable nature of the pain in FMF may cause pain catastrophizing. · The validity and reliability of Turkish Pain Catastrophizing Scale-Child (PCS-C) was shown for the first time in an adolescent age group. · Turkish PCS-C was introduced to clinical and research settings to evaluate the possible pain catastrophizing in FMF.

**Main Points**

- Unpredictable nature of the pain in FMF may cause pain catastrophizing.
- The validity and reliability of Turkish Pain Catastrophizing Scale-Child (PCS-C) was shown for the first time in an adolescent age group.
- Turkish PCS-C was introduced to clinical and research settings to evaluate the possible pain catastrophizing in FMF.

## Introduction

Familial Mediterranean fever (FMF) is the most common autosomal recessive auto-inflammatory disease characterized by recurrent episodes of fever and inflammation of serous membranes.^1,2^ Familial Mediterranean fever usually manifests during childhood, with most patients experiencing their initial attack before the age of 20^3^ Pain is the most reported symptom during attacks, along with fever in children with FMF.^1,4^ Familial Mediterranean fever attacks in children are usually resolved within days, while some patients may experience frequent episodes, ranging from 10 to 36 attacks per year.^5^ Even among those receiving advanced pharmacologic therapies (anti-IL-1 agents), attacks are still reported, with frequencies of approximately 6 to 12 episodes annually.^6^ Diffuse abdominal pain is the most reported type of pain, which is present in 84.6% of patients, followed by arthralgia (50.3%), myalgia (26.6%), chest pain (26.1%), and exertional leg pain (20.4%).^7^ Rarer painful manifestations include erysipelas-like erythema (6%-30%) and protracted febrile myalgia syndrome (~0.5%-1.6%).^8^ Patients with M694V and V726A genotypes, or the M694V/V726A genotype, were also reported to have a higher risk of pain attacks.^8^

Pain is not only a sensation but also a complex and subjective experience that is aligned with the biopsychosocial model. Pain severity reported by the patients may not necessarily correlate with the physical harm or tissue damage, due to altered peripheral and central pain pathways and/or emotional aspects of pain, especially in patients with long-standing diseases.^9,10^

Pain catastrophizing has emerged as a key area of interest in pain management and research in the last decades.^11,12^ The term defines the tendency to express the experience of pain in more excessive medians and is characterized as a negative cognitive-affective response to pain.^12^ This exaggeration may include repetitive/persisting thinking about pain experience (rumination), perceiving pain as more intense than it is (magnification), and/or feeling no influence/power on pain (helplessness).^13^

Pain Catastrophizing Scale-Child (PCS-C) was developed in Dutch from the adult version of the Pain Catastrophizing Scale^14^ to assess pain catastrophizing in children.^15^ Pain Catastrophizing Scale-Child was adapted into different languages including English,^16^ German,^17^ Spanish,^18^ and Brazilian Portuguese,^19^ and sufficient psychometric properties were demonstrated for each version.

Although pain catastrophizing is well investigated in many childhood and adulthood diseases with chronic/repetitive pain, no previous study has explored the effect of pain catastrophizing in childhood FMF, possibly due to the lack of a validated tool. Thus, the aim of this study was to investigate the psychometric properties (internal consistency, test-retest reliability, and convergent validity) of the Turkish version of the Pain Catastrophizing Scale-Child (PCS-C) in adolescents with FMF.

## Materials and Methods

### Study Design

This is an observational cross-sectional study. Ethical approval was obtained from the Ethics Committee of the Dokuz Eylül University Ethics Committee for Non-interventional Studies (date: July 25, 2013, protocol no: 2013/28-17). All participants and their legal guardians provided written informed consent before participation. The study adhered to the principles of the Declaration of Helsinki throughout all procedures.

### Participants

Adolescents with FMF who were under follow-up at the Pediatric Rheumatology Clinic of Dokuz Eylul University Nevvar&Salih İşgören Children’s Hospital were invited to participate between August 2013 and August 2014. The inclusion criteria were as follows: (a) being diagnosed with FMF according to the Turkish pediatric FMF criteria^20^ and (b) being between 13 and 18 years of age. Exclusion criteria were as follows: (a) having concomitant condition(s) that could affect pain perception, (b) a recent change in medication type/dosage, and/or (c) having a surgery/joint injection in the last 6 months prior to participation in the study.

A pilot study with 10 adolescents with FMF was performed to determine the preliminary effect sizes to calculate the necessary sample size. The G*Power v3.1.5.1 software (Heinrich Heine University, Düsseldorf, Germany) was used for the sample size calculation. The sample sizes were calculated separately for validity and reliability analyses to ensure that each aspect of the psychometric evaluation had sufficient statistical power.

An expected correlation coefficient of 0.579 (as observed between pain catastrophizing and quality of life outcomes in the pilot study), a null correlation coefficient of 0.3, a minimum power of 80%, and a maximum Type I error rate of 5% indicated that at least 66 participants were necessary for investigating the convergent validity of Turkish PCS-C.

An expected intraclass correlation coefficient (ICC) of 0.912 (as observed between the Turkish PCS-C test and retest outcomes in the pilot study) was used to determine the sample size for assessing test-retest reliability. Using a null correlation coefficient of 0.7, a minimum power of 80%, and a maximum type 1 error of 5%, it was calculated that at least 24 participants were necessary for the test-retest reliability analysis.

### Outcome Measures

#### Turkish Pain Catastrophizing Scale-Child:

Pain Catastrophizing Scale-Child (PCS-C) consists of 13 items assessing pain catastrophizing (e.g., When I am in pain, I keep thinking about how much it hurts). Each item is scored on a scale from 0 (not at all) to 4 (all the time), with the total score ranging from 0 to 52. Higher scores indicate a greater degree of pain catastrophizing.^15^ The PCS-C also provides 3 subscores corresponding to different aspects of pain catastrophizing: Rumination (items 8, 9, 10, 11), Magnification (items 6, 7, 13), and Helplessness (items 1, 2, 3, 4, 5, 12).^15^ The authors of the present study obtained official translation permission from the original developers of PCS-C and translated the scale into the Turkish language. However, Şentürk et al^21^ performed another validation study of the Turkish PCS-C in children with headaches. The authors used the Turkish adaptation of PCS-C (Appendix) developed for the present study. They reported good internal consistency, convergent validity, structural validity, and test-retest reliability in children with primary childhood headaches.^21^

#### Visual Analog Scale:

The pain intensity was measured using the Visual Analog Scale (VAS).^22^ A 100-millimeter horizontal line, with one end labeled as “No pain” and the other end labeled as “Worst pain imaginable,” was used. Participants were asked to mark on that line what corresponds to their pain at rest and during activities. The distances from the “no pain” end to the mark were measured and recorded in millimeters as VAS-rest and VAS-activity scores.

#### Pediatric Quality of Life Inventory Arthritis Module 3.0:

Quality of life was assessed using the Teenager Form (specialized for 13-18 years of age) of the Pediatric Quality of Life Inventory (PedsQL) 3.0 Arthritis Module.^23^ As FMF is considered a rheumatic disease, the PedsQL 3.0 Arthritis Module (representing rheumatic diseases) was preferred. The questionnaire consists of 22 items inquiring about “pain and suffering” (4 items), activities of daily livig (5 items), treatment (7 items), worry (3 items), and communication (3 items). Each item is rated on a 5-point Likert scale, with respondents indicating the frequency of the problem described in the item as experienced over the past month (0: no problem at all, 4: almost always). Item scores are reversed and converted into a score between 0 and 100 to calculate the total score, and higher scores indicate a better quality of life (QoL). The PedsQL 3.0 Arthritis Module has validity and reliability in the Turkish language.^23^

### Translation of Pain Catastrophizing Scale-Child into Turkish

The permission to adapt the scale into Turkish was obtained from the original authors on April 4, 2013. The scale was adapted using the method suggested by Beaton et al.^24^ Initially, 2 native Turkish speakers with advanced levels of English (1 health professional, 1 non-health professional) translated the scale into Turkish. The 2 translators then synthesized the forms into 1, with a third person to resolve disagreements. This version was then translated back into English by 2 native English speakers with advanced levels in Turkish. The forms were merged into one using the same procedure. Then, a commission consisting of researchers and translators reviewed the forms and finalized the Turkish version of the scale. The final version was assessed by 15 adolescents with FMF regarding language use, understandability, and clarity. As no major revisions were suggested, the final version of the Turkish PCS-C was used in the study (Appendix).

### Procedures

Adolescents with FMF were invited to participate in the study during their routine clinic visits. After providing informed consent, the physical characteristics (sex, age, height, weight, and body mass index) and disease-related characteristics (time since symptom onset, time since diagnosis, and colchicine use) of the participants were collected. Participants then completed VAS-rest, VAS-activity, and PedsQL. Turkish PCS-C was re-administered to 24 participants (36%) 2 weeks after the initial assessments by phone calls for test-retest reliability analysis.

### Statistical Analysis

SPSS 24.0 Software (IBM SPSS Corp.; Armonk, NY, USA) was used for all statistical analyses. Continuous data were expressed as mean ± SD or median IQR 25th/75th. The distribution of the continuous data was examined through histograms, Shapiro-Wilk test results, skewness/kurtosis values, and detrended Q-Q plots. *P* < .05 was considered statistically significant.

#### Internal Consistency:

A total Cronbach’s alpha value of at least 0.7, a minimum corrected item-total correlation of 0.4, and a minor decrease (<1%) in Cronbach’s alpha with the exclusion of an item was assumed to demonstrate the internal consistency of the Turkish PCS-C.^25^

#### Test-Retest Reliability:

Intraclass correlation coefficients (ICCs) with 95% confidence intervals (CI 95%) were calculated using a 2-way random effects model with absolute agreement (ICC 2,1) to investigate the test-retest reliability of the Turkish PCS-C. An ICC value of ≥ 0.7 was considered indicative of sufficient test-retest reliability.^25^

#### Convergent Validity:

Spearman’s rank correlation coefficients (r_s_) between the Turkish PCS-C and other instruments were calculated for convergent validity analysis. It was hypothesized that the Spearman correlation coefficients with other instruments r_s_ ≥ 0.3 indicate sufficient convergent validity for the Turkish PCS-C.^24^ The strength of correlations was categorized as follows: negligible (0 to 0.29), fair (0.30 to 0.49), moderate (0.50 to 0.69), strong (0.70 to 0.89), and very strong (0.90 to 1.00).^26^

## Results

The study was completed with 66 adolescents with FMF (age: 15.28 ± 1.54, 35 female). All participants were on colchicine with a median dosage of 1 mg (IQR 25th/75th= 1/1.5 mg). No participants were using biologic disease-modifying anti-rheumatic drugs. Physical characteristics, disease-related information, pain, QoL, and pain catastrophizing scores were summarized in Table 1.

### Internal Consistency

The initial internal consistency analysis resulted in a Cronbach’s alpha value of 0.926 (Table 2). Each item contributed to the total score sufficiently (item-total correlation > 0.4) except item 8 (item-total correlation = 0.206, Cronbach’s alpha with the exclusion of item 8 = 0.937) (Table 2). Thus, item 8 was excluded, and internal consistency was recalculated (Cronbach’s alpha= 0.937) (Table 3). Each item contributed adequately to the total score in the second analysis, as indicated by item-total correlations exceeding 0.4. Furthermore, the exclusion of an individual item did not result in a significant change in Cronbach’s alpha value (<1%), suggesting that all items were consistently aligned with the underlying construct. Further analyses (test-retest reliability, convergent validity) were conducted using the 12-item version of PCS-C.

### Test-Retest Reliability

Test-retest reliability analysis revealed that the Turkish PCS-C total score (ICC = 0.925) and the helplessness subscore (ICC = 0.950) exhibited excellent test-retest reliability (Table 4). The rumination (ICC = 0.725) and magnification (ICC = 0.752) subscores demonstrated good test-retest reliability (Table 4).

### Convergent Validity

Pain catastrophizing scores correlated significantly with pain at rest on a fair level (r_s _= 0.341 to 0.403, *P* < .05), with pain during activities on a fair to moderate level (r_s _= 0.357 to 0.536, *P* < .05), and with QoL scores on a fair to moderate level (r_s _= −0.498 to −0.551, *P* < .05, Table 5). The magnification subscore did not significantly correlate with pain at rest score (r_s _= 0.196, *P* = .115, Table 5).

## Discussion

The present study was performed to investigate the utility of the Turkish version of the PCS-C for assessing pain catastrophizing in adolescents with FMF. According to the results, the Turkish PCS-C demonstrated sufficient internal consistency, convergent validity, and test-retest reliability.

The final version of the Turkish PCS-C consisted of 12 items, and item 8 was excluded due to insufficient contribution to the internal consistency. This issue was also observed in previous studies that used English^16^ and Turkish^21^ versions of the PCS-C, though it was retained in other language versions.^15,17-19^ This discrepancy is likely due to variations in the translation process. Item 8 in the Dutch version “I anxiously want the pain to go away,” was changed into a more generic term, “I want the pain to go away,” in the English version. This likely caused all participants to give higher scores to item 8, as the item no longer questioned anxiety about the pain. Thus, item 8 lost its ability to inquire about the rumination of pain and solely became a question about pain. Given that Şentürk et al^21^ used the translation from the English version, the same low item contribution issue might have occurred. As a result, the present version of PCS-C has a maximum total score of 48, while the maximum rumination subscore is 12.

The present study is the first to investigate the reliability of subscores of PCS-C. The test-retest reliability for the total score and helplessness subscore of the Turkish PCS-C was excellent. However, rumination and magnification subscores were found to have relatively lower test-retest reliability scores, yet still had adequate test-retest reliability (ICC > 0.70). This might be related to the low number of items used in the calculation of these subscores (3 for each) compared to the helplessness subscore (6 items) and total score (12 items). The low number of items might have led to greater variability between test and retest outcomes of rumination and magnification subscores. Additionally, although absolute ICC values were higher than 0.7 for rumination and magnification subscores, the lower bounds in the 95% CI were lower than 0.7 for both subscores. Therefore, clinicians and researchers should be careful when interpreting magnification and rumination outcomes, as the reproducibility of these subscores might be lower for some children with FMF.

The solely convergent validity of the Turkish PCS-C was explored in the present study as no other available tool exists to assess the concurrent validity. Total and subscores of Turkish PCS-C demonstrated sufficient levels of correlation with pain levels and QoL of the participants. It was hypothesized that the PCS-C total score and its subscores would significantly correlate with pain and QoL (r_s _≥ 0.3) However, the correlations were between fair to moderate levels. Catastrophizing is often rooted in the long-term experience of pain and the emotional burden it creates; however, it represents a psychological process that is not identical to pain itself. Therefore, moderate correlations are not unexpected. The outcomes of 11 of the 12 correlation analyses confirmed the hypotheses, which is adequate to confirm the convergent validity of Turkish PCS-C according to the COSMIN Reporting guideline for studies on measurement properties of patient reported outcome measures (more than 75% of initial hypotheses were confirmed).^25^ Another point of interest about the correlation analyses is that the correlation coefficients between pain catastrophizing and QoL are higher than the correlation coefficients between pain catastrophizing and pain. These results indirectly demonstrate the negative effect of catastrophizing on QoL that is relatively independent of pain itself which was previously reported for other patient populations.

The presence of pain catastrophizing is considered a result of central sensitization and its biopsychosocial effect.^11,12^ Thus, children with rheumatic diseases are among the susceptible groups due to the chronic nature of the disease processes. A recent review reported that some degree of pain catastrophizing is present in patients with different rheumatic diseases of adulthood and childhood, including rheumatoid arthritis, spondyloarthritis, juvenile idiopathic arthritis, Sjögren’s syndrome, and systemic lupus erythematosus.^27^ Previously, adult participants with FMF were reported to have higher pain sensitization compared to a healthy population.^28^ Moreover, the unpredictable nature of pain attacks is likely to contribute to pain catastrophizing. However, to the authors’ knowledge, no previous study has investigated pain catastrophizing in individuals with FMF. Although this study did not employ a healthy control group, the results obtained for the Turkish PCS-C total scores were comparable to the mean scores reported for other rheumatic diseases. Thus, a future study comparing pain catastrophizing in children/adults with FMF to age/sex-matched healthy controls would help to demonstrate the extent of pain catastrophizing.

The most important limitation of the present study was not being able to investigate the structural validity of Turkish PCS-C using classical test theory or item-response theory due to insufficient sample size. A factor analysis with an insufficient number of participants would have led to a misleading factorial structure; thus, the same 3-factor structure suggested by the original authors has been used.^15^ Additionally, only adolescents with FMF were included. This limited the generalizability of the results of the present study.

The results of the present study suggest that the Turkish PCS-C is a valid and reliable tool to evaluate pain catastrophizing in adolescents with FMF. The Turkish PCS-C can be utilized in clinical and research settings to evaluate the possible pain catastrophizing, which is often an overlooked concept in individuals with chronic pain.

## Figures and Tables

**Table 1.** Patient Characteristics (n = 66)

	n/n, Mean ± SD or Median (IQR 25/75)
Sex (female/male)	35/31
Age (years)	15.3 ± 1.5
BMI (kg/m^2^)	20.83 ± 3.25
Time since the onset of symptoms (months)	120 (60/144)
Time since diagnosis (months)	96 (25.5/132)
Colchicine dosage (mg)	1 (1/1.5)
Pain	
VAS-rest (0-100 mm)	29.0 (3.8/54.8)
VAS-activity (0-100 mm)	45.5 (10.8/76.3)
Quality of Life	
PedsQL (score:0-100)	77.8 (63.5/89.2)
Pain Catastrophizing	
Turkish PCS-C Rumination (score: 0-16)	5.8 ± 3.5
Turkish PCS-C Magnification (score: 0-12)	4.0 ± 2.9
Turkish PCS-C Helplessness (score: 0-20)	9.1 ± 6.4
Turkish PCS-C Total (score: 0-48)	18.8 ± 11.8

IQR 25/75, interquartile range between the 25th and 75th percentiles; kg, kilogram; m, meter; mg, milligram; mm, millimeter; n, number of participants; PCS-C, Pain Catastrophizing Scale-Child; PedsQL, Pediatric Quality of Life Inventory; VAS, Visual Analog Scale.

**Table 2.** Initial Internal Consistency (n = 66)

	Corrected Item-Total Correlation	Cronbach’s Alpha If Item Deleted
Item 1	0.499	0.925
Item 2	0.688	0.919
Item 3	0.790	0.915
Item 4	0.824	0.914
Item 5	0.719	0.918
Item 6	0.737	0.917
Item 7	0.611	0.922
Item 8	0.206	0.937
Item 9	0.765	0.916
Item 10	0.746	0.917
Item 11	0.465	0.928
Item 12	0.808	0.914
Item 13	0.684	0.919
Turkish PCS-C Cronbach’s alpha = 0.926		

n, number of participants; PCS-C, Pain Catastrophizing Scale-Child.

**Table 3.** Internal Consistency Following Exclusion of Item 8 (n = 66)

	Corrected Item-Total Correlation	Cronbach’s Alpha If Item Deleted
Item 1	0.591	0.936
Item 2	0.751	0.931
Item 3	0.783	0.930
Item 4	0.813	0.929
Item 5	0.772	0.930
Item 6	0.763	0.930
Item 7	0.545	0.938
Item 9	0.785	0.929
Item 10	0.790	0.929
Item 11	0.553	0.939
Item 12	0.849	0.927
Item 13	0.644	0.935
Turkish PCS-C Cronbach’s alpha = 0.937		

n, number of participants; PCS-C, Pain Catastrophizing Scale-Child.

**Table 4.** Test-Retest Reliability of Turkish Pain Catastrophizing Scale-Child (n = 24)

	Test (Mean ± SD)	Retest (Mean ± SD)	ICC (95% CI)
Total Score (score = 0-48)	16.6 ± 11.7	16.9 ± 10.0	0.925 (0.840-0.968)*
Rumination (score = 0-12)	4.8 ± 3.4	5.0 ± 2.7	0.725 (0.460-0.871)*
Magnification (score = 0-12)	3.6 ± 2.7	3.8 ± 2.3	0.752 (0.506-0.885)*
Helplessness (score = 0-24)	8.2 ± 6.5	8.1 ± 6.2	0.950 (0.887-0.978)*

CI, confidence interval; ICC, intraclass correlation coefficient; n, number of participants.

*P < .05.

**Table 5.** Convergent Validity of Turkish Pain Catastrophizing Scale-Child (n = 66)

		VAS-Rest	VAS-Activity	PedsQL
Rumination (score = 0-12)	r_s_	0.341*	0.422*	−0.498*
	*P*	.005	<.001	<.001
Magnification (score = 0-12)	r_s_	0.196	0.357*	−0.509*
	*P*	.115	.003	<.001
Helplessness (score = 0-24)	r_s_	0.403*	0.573*	−0.528*
	*P*	.001	<.001	<.001
Total (score = 0-48)	r_s_	0.373*	0.536*	−0.551*
	*P*	.002	<.001	<.001

mm, millimeter; n, number of participants; PedsQL, Pediatric Quality of Life Inventory; r_s_, Spearman’s rank correlation coefficient; VAS, Visual Analog Scale.

*r_s _≥ 0.3 and *P* < .05.

## Data Availability

The data that support the findings of this study are available on request from the corresponding author.
